# CSN5 inhibition triggers inflammatory signaling and Rho/ROCK-dependent loss of endothelial integrity

**DOI:** 10.1038/s41598-019-44595-4

**Published:** 2019-05-31

**Authors:** Jisca Majolée, Manon C. A. Pronk, Kin K. Jim, Jan S. M. van Bezu, Astrid M. van der Sar, Peter L. Hordijk, Igor Kovačević

**Affiliations:** 1 Amsterdam UMC, Vrije Universiteit Amsterdam, Department of Physiology, Amsterdam Cardiovascular Sciences, De Boelelaan 1117, 1081HZ Amsterdam, The Netherlands; 2 Amsterdam UMC, Vrije Universiteit Amsterdam, Department of Medical Microbiology and Infection Control, Amsterdam Infection and Immunity Institute, De Boelelaan 1117, 1081HZ Amsterdam, The Netherlands

**Keywords:** RHO signalling, Vascular diseases

## Abstract

RhoGTPases regulate cytoskeletal dynamics, migration and cell-cell adhesion in endothelial cells. Besides regulation at the level of guanine nucleotide binding, they also undergo post-translational modifications, for example ubiquitination. RhoGTPases are ubiquitinated by Cullin RING ligases which are in turn regulated by neddylation. Previously we showed that inhibition of Cullin RING ligase activity by the neddylation inhibitor MLN4924 is detrimental for endothelial barrier function, due to accumulation of RhoB and the consequent induction of contractility. Here we analyzed the effect of pharmacological activation of Cullin RING ligases on endothelial barrier integrity *in vitro* and *in vivo*. CSN5i-3 induced endothelial barrier disruption and increased macromolecule leakage *in vitro* and *in vivo*. Mechanistically, CSN5i-3 strongly induced the expression and activation of RhoB and to lesser extent of RhoA in endothelial cells, which enhanced cell contraction. Elevated expression of RhoGTPases was a consequence of activation of the NF-κB pathway. In line with this notion, CSN5i-3 treatment decreased IκBα expression and increased NF-κB-mediated ICAM-1 expression and consequent adhesion of neutrophils to endothelial cells. This study shows that sustained neddylation of Cullin RING-ligases leads to activation the NF-κB pathway in endothelial cells, elevated expression of RhoGTPases, Rho/ROCK-dependent activation of MLC and disruption of the endothelial barrier.

## Introduction

Endothelial barrier function controls vascular integrity and is essential for normal physiology. Both under resting conditions, and following agonist-mediated stimulation, endothelial integrity is regulated at the level of both the actin cytoskeleton and cell-cell contacts^1,2^. Actin dynamics are regulated by members of the family of RhoGTPases. Rac1 and Cdc42 drive actin polymerization and formation of membrane protrusions which enhance endothelial barrier function, while RhoA induces formation of actin stress fibers and cell contraction which disrupts endothelial barrier function^3–5^. Besides being regulated at the level of guanine nucleotide binding^6^, RhoGTPases undergo post-translational modifications such as ubiquitination, which control their activity, localization and expression^7–9^.

Protein ubiquitination refers to the covalent attachment of the 76 amino acid ubiquitin protein to lysine residues in substrates, and is classically known as part of the degradation pathway called the ubiquitin proteasome system (UPS). Besides protein degradation, ubiquitination drives internalization and trafficking of proteins, regulating, a.o., inflammatory signaling, autophagy and DNA repair^10^. In the first step of ubiquitination, ubiquitin is activated by binding to a ubiquitin-activating enzyme (E1). Activated ubiquitin is subsequently attached to a ubiquitin-conjugating enzyme (E2) and covalently linked to a lysine residue of the substrate protein by a ubiquitin protein ligase (E3)^11^. E3 ligases are divided into two classes based on the presence of the Homologous to the E6-AP Carboxyl Terminus (HECT) domain or a Really Interesting New Gene (RING) domain^12^. Members of the large group of RING-E3 ligases serve as scaffolds that bring substrates and the E2 conjugating enzyme together for ubiquitin conjugation. Cullins are RING-ligases that undergo a conformational change upon neddylation, the covalent attachment of the ubiquitin-like protein Nedd8. This neddylation-induced conformational change of Cullin promotes polyubiquitination of the substrate and prevents the binding of the inhibitor CAND1^13,14^. This process is reversed by de-neddylation, in which Nedd8 is removed by isopeptidase activity of the COP9 Signalosome (CSN)^15^.

The effect of the COP9 signalosome, and more specifically of CSN5, on the activity of Cullin E3 ligases has been investigated both *in vivo*^16,17^ and *in vitro*^18,19^. In addition to increased substrate ubiquitination, neddylation of Cullins has been shown to induce auto-ubiquitination and degradation of the CRL complex. Besides exhibiting deneddylation activity, the CSN is associated with the deubiqutinase Ubp12/USP15, hereby increasing the stability of Cullins and their associated adaptor proteins^19^. Several years ago, the neddylation inhibitor MLN4924 was developed, a selective inhibitor of the Nedd8-activating enzyme (NAE) which is currently tested in clinical trials for cancer treatment^20–22^. MLN4924 also protects against pulmonary fibrosis by inhibition of inflammation in animals and in human cell-lines^23^. Aiming to further explore the therapeutic window of modulators of the UPS, a COP9 signalosome inhibitor was developed by Schlierf *et al*.^24^.

This compound, named CSN5i-3, inhibits the activity of CSN5, which forms the catalytic core of the COP9 signalosome. As a result, deneddylation of Cullins is inhibited. Whereas MLN4924 treatment results in accumulation of the deneddylated, inactive isoform of Cullins, CSN5i-3 stabilizes Cullin neddylation and –activity. In functional assays, CSN5i-3 inhibits differentiation of cancer cells *in vitro* and inhibits tumor growth *in vivo*^24^.

Recently, we showed that inhibition of the Cullin-RING E3 ubiquitin ligases in endothelial cells, either by MLN4924 or shRNA-mediated knockdown of Cullin-3, increased the expression of the RhoGTPase RhoB. This was accompanied by the RhoB-dependent formation of F-actin stress fibers, contraction and a loss of endothelial barrier function^9^. Since we found that the general inhibition of Cullin neddylation was detrimental for endothelial barrier function we set out to investigate the physiological relevance of improved Cullin neddylation on endothelial barrier function and in particular on the activity and expression of RhoGTPases. Therefore, we investigated the effect of CSN5i-3 on cultured primary Human Umbilical Vein Endothelial Cells (HUVECs) *in vitro* and in zebrafish embryos *in vivo*.

In HUVECs we observed decreased endothelial barrier function and increased macromolecular passage upon application of CSN5i-3. In line with these findings, treatment of zebrafish embryos with this compound increased vascular permeability. In subsequent analysis, we observed that CSN5i-3 activated the Rho/Rho Kinase (ROCK) signaling pathway and induced consequent cell contraction. This was mainly due to increased *de novo* Rho protein synthesis which in turn was caused by the activation of the NFkB pathway in CSN5i-3 treated endothelial cells. Thus, pharmacological activation of Cullin Ring Ligases induces an NFkB-mediated inflammatory response in endothelial cells accompanied by a marked loss of junctional integrity.

## Results

### Inhibition of the COP9 signalosome disrupts endothelial barrier integrity

**Figure 1:**
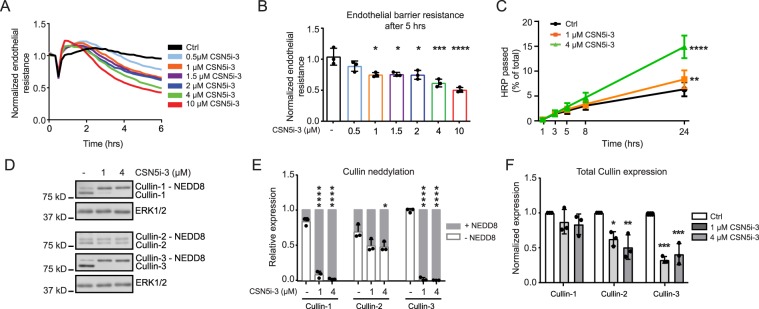
CSN5i-3 disrupts endothelial barrier integrity and differentially affects Cullin-1, -2 and -3. (**A**) Time course of normalized endothelial resistance of HUVEC monolayers during CSN5i-3 treatment in different concentrations. (**B**) Normalized endothelial resistance at timepoint 5 hours after CSN5i-3 treatment in different concentrations. *p < 0.05, ***p < 0.001, ****p < 0.0001 compared to control in Dunnet’s post-hoc test of one-way ANOVA (n = 4). (**C**) Macromolecule passage (Horseradish peroxidase, HRP) across HUVEC monolayers during CSN5i-3 treatment **p < 0.01, ****p < 0.0001 compared to control in Tukey’s post-hoc test of two-way repeated measures ANOVA (n = 3 independent experiments). (**D**) Western blot analysis of Cullin-1, Cullin-2 and Cullin-3 after 5 hours of CSN5i-3 treatment (1 μM or 4 μM). ERK1/2 was used as loading control. Blot images were cropped for clarity of presentation (full blots are in Supplemental Fig. 3). (**E**) Quantification of neddylated versus unneddylated Cullin-1, Cullin-2 and Cullin-3 after CSN5i-3 treatment. *p < 0.05, ***p < 0.001 compared to control in Dunnett’s post-hoc test of one-way ANOVA (n = 3 independent experiments). (**F**) Quantification of total Cullin-1, Cullin-2 and Cullin-3 levels after CSN5i-3 treatment. *p < 0.05, **p < 0.01, ***p < 0.001 compared to control in Dunnett’s post-hoc test of one-way ANOVA (n = 3).

CSN5i-3 was designed as an inhibitor of the COP9 signalosome which mediates removal of Nedd8 from the Cullin subunit of Cullin-RING ubiquitin ligases, thus inactivating the complex^24^. To test the effect of CSN5i-3 on endothelial barrier function, we added the compound in different concentrations to confluent primary HUVECs. Electrical cell-substrate impedance sensing (ECIS) was used to quantify changes in endothelial barrier function in real time. Within 1 hour after addition of CSN5i-3 we observed a small increase in barrier function, after which the integrity of the endothelial barrier decreased. This reduction in endothelial barrier was dose-dependent (Fig. 1A). 1 μM CSN5i-3 or higher induced a significant attenuation of endothelial barrier function at 5 hours after addition (Fig. 1B). In addition to reduced resistance of the endothelial barrier, we observed significant barrier disruption by CSN5i-3 in HRP-leakage experiments after prolonged stimulation (>5 hours) (Fig. 1C). Cytotoxicity of CSN5i-3 was reported in cancer cell lines by Schlierf *et al*. after 72 hours of treatment^24^. To investigate if loss of endothelial barrier integrity was caused by increased apoptosis, we analyzed caspase-3/7 activity in cells treated with CSN5i-3 for 5 and 24 hours (Supplemental Fig. 1). After 5 hours of CSN5i-3 treatment (1 and 4 uM), caspase-3/7 activity was not induced, suggesting that initial disruption of endothelial integrity is not caused by apoptosis. However, we observed increased caspase-3/7 activity after 24 hours of treatment with CSN5i-3 which implicates contribution of apoptosis to the increase of HRP leakage at later timepoints. In subsequent experiments we examined the effect of CSN5i-3 on Cullin-1, Cullin-2 and Cullin-3 neddylation in endothelial cells using immunoblotting (Fig. 1D). We observed a significant mobility shift marking the neddylated isoforms for Cullin-1 and Cullin-3, while this shift was less pronounced for Cullin-2 (Fig. 1D,E). Moreover, CSN5i-3 induced a decrease in total Cullin-2 and Cullin-3 expression, while the levels of Cullin-1 remained unaltered (Fig. 1F). From these data we conclude that CSN5i-3 stabilizes Cullin 1–3 neddylation and differentially affects their expression. Together, this results in a dose-dependent, significant disruption of the endothelial barrier.

### CSN5i-3 induces expression and activity of RhoGTPases

**Figure 2:**
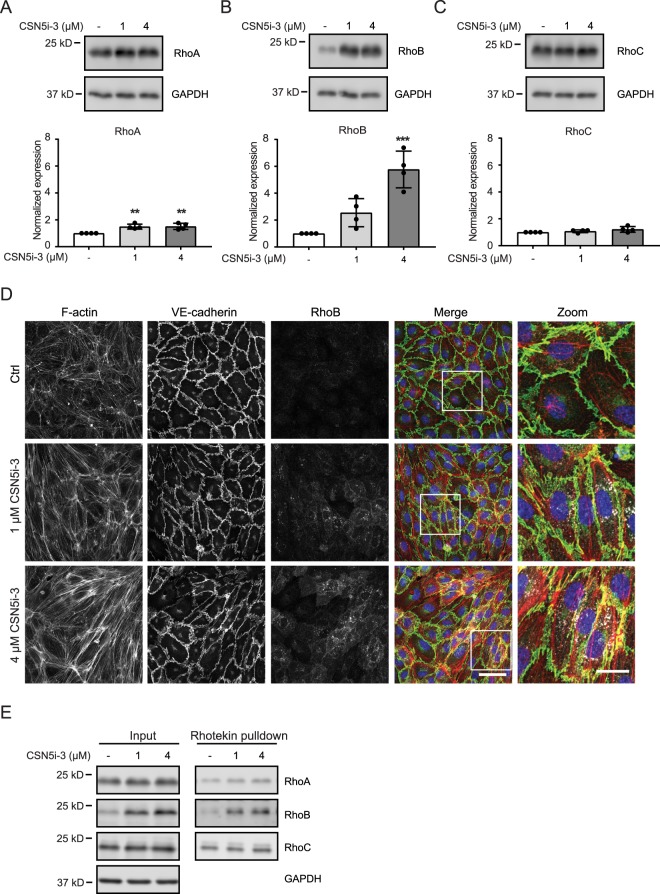
CSN5i-3 treatment induces expression of RhoB in endothelial cells. (**A**–**C**) Western blot analysis of RhoA (**A**), RhoB (**B**), RhoC (**C**) expression after 5 hours of CSN5i-3 (1 μM or 4 μM) treatment. GAPDH was used as loading control. Blot images were cropped for clarity of presentation (full blots are in Supplemental Fig. 4). *p < 0.05, **p < 0.01 compared to control in Dunnett’s post-hoc test of one-way ANOVA (n = 4). (**D**) Evaluation of RhoB (white), F-actin (red) and VE-cadherin (green) in control versus CSN5i-3 treated cells counterstained with DAPI (blue). Representative images of n = 3 experiments. Scale bar represents 50 μm in overview images and 20 μm in zoomed images. (**E**) Rhotekin pulldown using lysates of HUVECs treated with CSN5i-3 (1 or 4 μM) for 5 hours followed by Western blot analysis for RhoA, RhoB and RhoC. GAPDH was used as loading control. Blot images were cropped for clarity of presentation (full blots are in Supplemental Fig. 5).

Previously, we showed that inhibition of Cullin-3 activity resulted in increased expression of RhoB due to a reduction of RhoB-ubiquitination with only a modest effect on RhoA^9^. Therefore, we tested the effect of CSN5i-3-mediated Cullin activation on the protein expression of RhoA, RhoB and RhoC. Five hours after addition of 1 and 4 μM CSN5i-3, RhoA and RhoC protein levels were slightly increased (Fig. 2A,C) with RhoB expression levels 2- and 5-fold increased, respectively (Fig. 2B). Previously, we showed that RhoB ubiquitination controls its subcellular localization in endothelial cells^9^ and we therefore analyzed RhoB localization by immunofluorescent staining. Treatment with 1 μM CSN5i-3 induced the upregulated RhoB to localize both to intracellular vesicles and the cytoplasm. This was accompanied by stress fiber formation and a more discontinuous staining for Vascular Endothelial (VE)-cadherin (Fig. 2D). Higher concentration of CSN5i-3 (4 μM) induced similar, but more pronounced effects on RhoB expression and endothelial cell morphology (Fig. 2D). To test if the induction of RhoGTPase expression and change in cell morphology, induced by CSN5i-3, was accompanied by increased activity of RhoGTPases, we performed Rhotekin pulldowns. We found that CSN5i-3 strongly increased the activity of RhoB, with limited effects on RhoA and RhoC, directly correlating with the differential effects on their expression (Fig. 2E). Together, these data indicate that treatment of endothelial cells with CSN5i-3 induces the expression of RhoGTPases, primarily RhoB, and the formation of actin stress fibers.

### Endothelial barrier disruption by CSN5i-3 is ROCK-mediated

**Figure 3:**
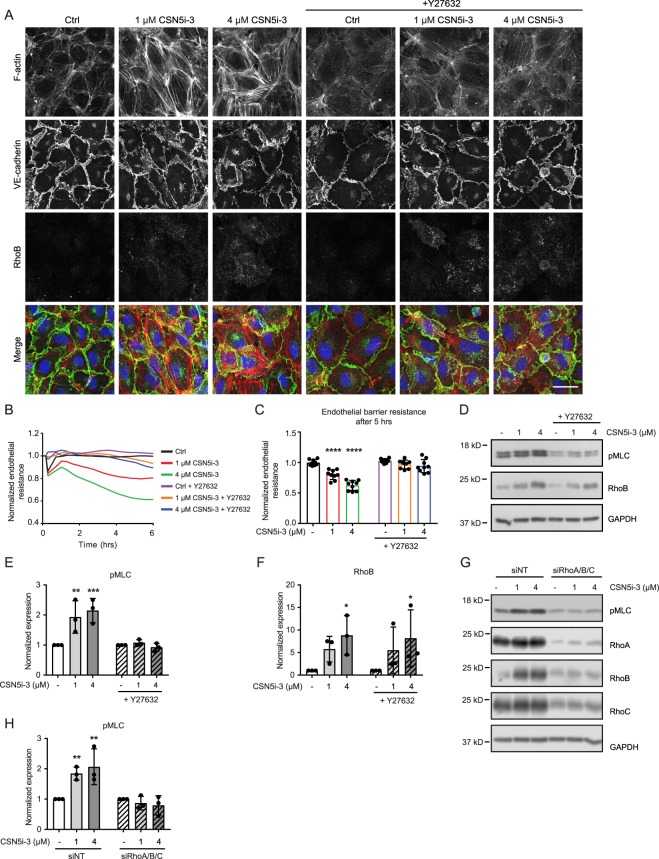
CSN5i-3 effect on endothelial barrier can be counteracted by inhibition of Rho/ROCK pathway. (**A**) Evaluation of effect on F-actin (red), VE-cadherin (green) and RhoB (white) in control versus CSN5i-3- (1 and 4 μM) and Y27632-treated cells counterstained with DAPI (blue). Representative images of n = 3 experiments. Scale bar represents 30 μm. (**B**) Time course of normalized endothelial resistance of HUVEC monolayers during CSN5i-3 treatment following pre-incubation with Y27632. (**C**) Normalized endothelial resistance after 5 hours of CSN5i-3 treatment with or without pre-treatment of Y27632. ***p < 0.001, ****p < 0.0001 in Dunnet’s post-hoc test of two-way ANOVA (n = 3). (**D–F**) Western blot analysis and quantification of pMLC (**D**,**E**) and RhoB (**D**,**F**) after 5 hours of CSN5i-3 treatment (1 μM or 4 μM) with or without pre-treatment with Y27632. GAPDH was used as loading control. Blot images were cropped for clarity of presentation (full blots are in Supplemental Fig. 6). **p < 0.01 in Dunnet’s post-hoc test of two-way ANOVA (n = 3). (**G**,**H**) Western blot analysis and quantification of pMLC after 5 hours of CSN5i-3 treatment (1 μM and 4 μM) of combined RhoA, RhoB and RhoC knockdown cells. GAPDH was used as loading control. Blot images were cropped for clarity of presentation (full blots are in Supplemental Fig. 7). **p < 0.01 in Dunnet’s post-hoc test of two-way ANOVA (n = 3).

Cell contraction underlies endothelial barrier disruption and is initiated by the activation of Rho GTPases and their downstream effector Rho-kinase (ROCK)^25^. We previously showed that RhoB is important for endothelial cell contraction^4^. Immunofluorescent staining of HUVECs treated with CSN5i-3 showed increased actin stress fiber formation, which is likely mediated by Rho/ROCK activation and Myosin Light Chain (MLC) phosphorylation^26^. Treatment with the ROCK inhibitor Y27632 of otherwise unstimulated cells, reduced stress fiber formation while VE-cadherin staining showed stable, honeycomb-like cell-cell contacts (Fig. 3A). Addition of CSN5i-3 promoted stress fiber formation while the VE-cadherin showed a more jagged distribution, characteristic for remodeling adherens junctions^27^. Pre-treatment of cells with Y27632 clearly abrogated CNS5i-3 –induced stress fiber formation and stabilized VE-cadherin-positive junctions (Fig. 3A). In accordance with this finding, pre-treatment of HUVECs with Y27632 prevented the barrier disruptive effect of CSN5i-3 as shown in ECIS experiments (Fig. 3B,C). Furthermore, CSN5i-3 increased MLC phosphorylation as shown by immunoblot analysis, and this was completely abolished by pre-treating the cells with Y27632 (Fig. 3D,E). Importantly, pre-treatment with Y27632 did not impair CSN5i-3-induced expression of RhoB protein (Fig. 3A,D,F).

In addition to pharmacological inhibition of the Rho/ROCK signaling pathway, we performed a siRNA-mediated knockdown of RhoA, RhoB and RhoC to establish the contribution of these Rho GTPases to the morphological changes caused by CSN5i-3. Individual depletion of RhoA, RhoB or RhoC did not effectively prevent phosphorylation of MLC by CSN5i-3 (Supplemental Fig. 2). This is likely because these GTPases regulate each other’s expression such that loss of one drives increased expression of the other^4^, which in turn leads to functional compensation^4^. Therefore, we performed a triple knockdown of all three GTPases simultaneously and analyzed CSN5i-3-induced MLC phosphorylation. We found that only the combined depletion of RhoA, -B and -C effectively abrogated CSN5i-3-induced MLC phosphorylation. In conclusion, our data indicate that the barrier disruption caused by CSN5i-3 is mediated by activation of RhoGTPase/ROCK signaling leading to increased MLC phosphorylation and subsequent cell contraction.

### CSN5i-3 increases RhoB expression partially via transcriptional upregulation

**Figure 4:**
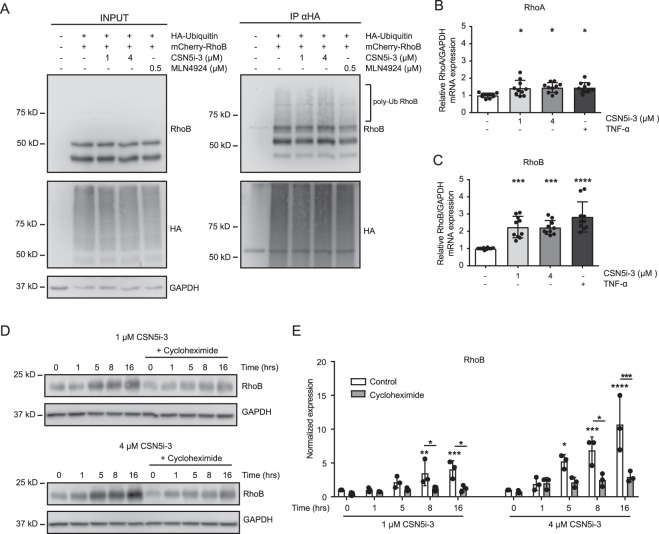
CSN5i-3- induces transcription of RhoB mRNA. (**A**) HEK293T cells were cotransfected with mCherry-RhoB and HA-ubiquitin and treated with CSN5i-3 (1 and 4 μM) or MLN4924 for five hours, with addition of MG132 for the last four hours. Next, HA-ubiquitin was immunoprecipitated under denaturing conditions. Samples were analyzed by western blot for presence of RhoB and ubiquitin using RhoB and HA antibodies, respectively. GAPDH was used as loading control. Blot images were cropped for clarity of presentation (full blots are in Supplemental Fig. 8). (**B**,**C**) RNA expression, determined by qPCR, of RhoA (**B**) and RhoB (**C**) from HUVECs lysates after 5 hours of treatment with CSN5i-3 (1 and 4 μM) or 10 ng/ml TNF-α *p < 0.05, ***p < 0.001, ****p < 0.0001 after Dunnet’s post hoc analysis of one-way ANOVA (n = 3). (**D**–**E**) Western blot analysis and quantification of RhoB expression after CSN5i-3 treatment (1 and 4 μM) for 0, 1, 5, 8 and 16 hours, with or without pre-treatment of 0.5 μg/ml cycloheximide. Blot images were cropped for clarity of presentation (full blots are in Supplemental Fig. 9). *p < 0.05, **p < 0.01, ***p < 0.001, ****p < 0.0001 after Dunnet’s post hoc analysis of two-way ANOVA (n = 3).

We previously showed that inhibition of Cullin RING ligases by MLN4924 decreased RhoB ubiquitination, hereby interfering with RhoB degradation^9^. An *in vivo* ubiquitination assay in HEK293T cells showed that CSN5i-3 treatment, in contrast to MLN4924, did not significantly change the ubiquitination state of RhoB (Fig. 4A). To test a role for *de novo* protein synthesis, we analyzed the effect of CSN5i-3 on the mRNA expression of RhoA and RhoB. Addition of CSN5i-3 (1 and 4 μM) or stimulation with TNF-α, as a positive control, only slightly increased RhoA mRNA expression (Fig. 4B). However, the mRNA expression of RhoB in response to treatment with CSN5i-3 (1 and 4 μM) was induced 2.2-fold, with the TNF-α-mediated induction of RhoB mRNA being 2.8-fold (Fig. 4C). In accordance with this, inhibition of mRNA translation by cycloheximide significantly impaired induction of RhoB protein by CSN5i-3 (Fig. 4D,E). These results suggest that the CSN5i-3-mediated increase in RhoB expression is, rather than to altered ubiquitination, mainly due to enhanced *de novo* synthesis of RhoB protein.

### Inhibition of the COP9 signalosome activates NF-κB and enhances ICAM expression and leukocyte adhesion

**Figure 5:**
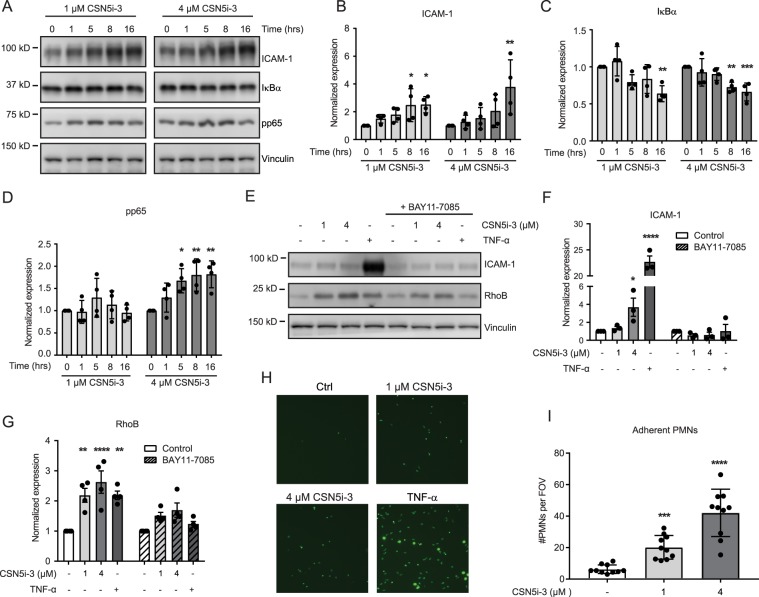
CSN5i-3 increases RhoB levels by activation of the NF-κB pathway. (**A**–**D**) Western blot analysis and quantification of ICAM-1 (**B**), IκBα (**C**) and pp65 (**D**) expression after CSN5i-3 treatment (1 and 4 μM) for 0, 1, 5, 8 and 16 hours. Vinculin was used as loading control. Blot images were cropped for clarity of presentation (full blots are in Supplemental Fig. 10). *p < 0.05, **p < 0.01, ***p < 0.001 after Dunnett’s post-hoc analysis of one-way ANOVA (n = 4). (**E**–**G**,**H**) Western blot analysis and quantification (bar graphs) of ICAM-1 (**F**) and RhoB (**G**) expression after 5 hours of CSN5i-3 (1 μM or 4 μM) or TNF-α (10 ng/mL) in combination with BAY11-7085 treatment (10 μM). Vinculin was used as loading control. Blot images were cropped for clarity of presentation (full blots are in Supplemental Fig. 11). *p < 0.05, **p < 0.01 ***p < 0.001 after Sidaks post-hoc analysis of two-way ANOVA. (n = 3 for ICAM-1, n = 4 for RhoB). (**H**) Representative pictures of fluorescently labeled PMN adherent to a HUVEC monolayer treated with CSN5i-3 or TNF-α. (**I**) Quantification of PMNs per field of view after CSN5i-3 treatment. ***p < 0.001, ****p < 0.0001 after Dunnet post-hoc analysis of one-way ANOVA (n = 4 separate experiments with 10 fields of view per experiment).

Induction of RhoB mRNA by TNF-α was previously described^9,28,29^. Since we found that CSN5i-3 induced RhoB mRNA synthesis similar to TNF-α, and because Cullin-RING ligases have been implicated in TNF-α mediated NF-κB activation^30^, we hypothesized that CSN5i-3 increases RhoB mRNA expression via NF-κB. In resting cells, the cytosolic NFκB p65 subunit is bound to members of the family of inhibitory IκB proteins. Degradation of IκB occurs upon their phosphorylation and subsequent ubiquitination by βTRCp-Cullin-1, followed by proteasomal degradation^30^. Degradation of IκB allows the p65-NFκB complex to translocate to the nucleus and activate transcription of its target genes, including the leukocyte adhesion molecule ICAM-1. Treatment of HUVECs with CSN5i-3 resulted in significantly reduced IκBα expression (Fig. 5A,C). Conversely, phosphorylation of the p65 subunit of NFκB was significantly increased only after prolonged CSN5i-3 (4 μM) treatment (Fig. 5A,D). In addition, we the expression of ICAM-1 was significantly increased (Fig. 5A,B). To confirm the role of the NF-κB pathway in the CSN5i-3-induced upregulation of RhoB, we applied the specific IκB phosphorylation inhibitor BAY11-7085 in combination with CSN5i-3^31^. Treatment of HUVECs with BAY11-7085 significantly reduced both the TNF-α and CSN5i-3-induced increase in RhoB levels (Fig. 5E,G). Also, TNF-α and CSN5i-3-induced ICAM-1 expression was completely blocked by BAY11-7085 (Fig. 5E,F).

Since CSN5i-3 increased ICAM-1 expression, we tested if this increase is physiologically relevant in a leukocyte adhesion assay. We treated HUVEC monolayers with CSN5i-3 or TNF-α and analyzed the adhesion of polymorphonuclear neutrophils (PMN) which depend on ICAM-1 for strong adhesion. PMN adhesion was dose dependently increased in endothelial cells treated with CSN5i-3 as compared to control cells (Fig. 5H,I). TNF treatment increased PMN adhesion further, in good agreement with its stronger effect on ICAM-1 upregulation (Fig. 5E,F,H). In conclusion, our data indicate that CSN5i-3 induces degradation of IκBα, most probably via activation of Cullin-1 RING ligase^30^, resulting in induction of the pro-inflammatory NF-κB pathway. This drives the increased expression of RhoB and ICAM-1, resulting in a loss of endothelial barrier function and increased leukocyte adhesion, respectively.

### CSN5i-3 promotes vascular leakage in zebrafish embryos

**Figure 6:**
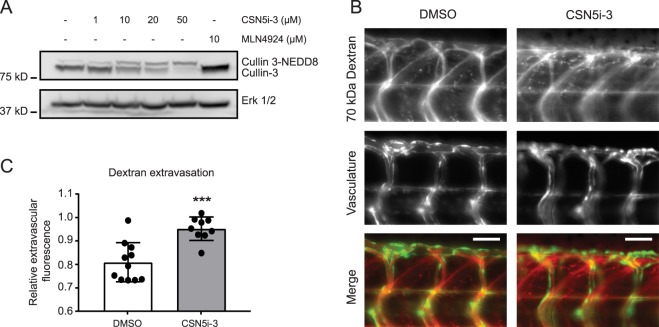
CSN5i-3 increases vascular leakage *in vivo*. (**A**) Zebrafish *Tg(Fli1:GFP)y1* casper embryos were treated with 1, 10, 20 and 50 μM CSN5i-3, 10 μM MLN4924 or solvent (DMSO 0.5%) for 48 hours from 24 hpf onward. Embryos were lysed and western blot analysis of Cullin-3 was performed. ERK 1/2 was used as loading control. Blot images were cropped for clarity of presentation (full blots are in Supplemental Fig. 12). (**B**) Representative images of vascular leakage in control vs CSN5i-3 treated zebrafish embyros. Zebrafish embryos were treated with 50 μM CSN5i-3 or solvent (DMSO 0.5%) from 1 dpf onward for 72 hours. 70 kDa TMR-dextran was injected at the intersection of the common cardinal vein, the posterior cardinal vein and the primary head sinus to visualize vascular leakage. 70 kDa Dextran is in red, vasculature in green. Scale bar represents 50 μm. (**C**) Quantification of relative extravascular fluorescence from 0.5% DMSO (n = 11) or 50 μM CSN5i-3 (n = 9) treated fish. For each embryo, the fluorescence intensity of the dextran was measured in four intersegmental vessels and three intervascular areas between the intersegmental vessels. The average of the dextran fluorescence in the intervascular areas was normalized to the average dextran fluorescence inside the vessels. Obtained values represent the ratio of extravasated dextran compared to dextran inside the vessels. ***p < 0.001 compared to control in student’s t-test.

CSN5i-3 treatment disrupted endothelial barrier integrity *in vitro* in primary human endothelial cells. To confirm this finding *in vivo*, we examined the effect of CSN5i-3 on zebrafish (*Danio rerio*) vascular integrity. At 24 hours post fertilization (hpf), CSN5i-3 was added to the swimming water of *Tg(Fli1:GFP)*^*y1*^ casper zebrafish embryos for 48–72 hours. In a dose-response experiment, we found that 50 μM CSN5i-3 was required to induce full neddylation of zebrafish Cullin-3 (Fig. 6A). Similar to our observation in *in* vitro experiments, total Cullin-3 levels were decreased in zebrafish embryos treated with CSN5i-3. As a negative control, zebrafish embryos were treated with 10 μM MLN4924, and as expected, we observed a clear shift to the de-neddylated form of Cullin-3 (Fig. 6A). To assess whether CSN5i-3 induces vascular leakage in the zebrafish embryos, 70 kDa TMR Dextran was injected directly into the bloodstream. We performed live fluorescent imaging of the zebrafish embryos 20 minutes after injection of the dextran (Fig. 6B) and quantified the relative dextran extravasation (Fig. 6C). We observed a significant increase in dextran leakage from the intersegmental vessels in 4 days post fertilization (dpf) zebrafish embryos treated with CSN5i-3 (Fig. 6C). In conclusion, our data indicate that CSN5i-3 treatment induces *in vivo* vascular leakage.

## Discussion

Here we show that inhibition of CSN5, the catalytic component of the COP9 signalosome, disrupts endothelial barrier function *in vitro* and *in vivo*. The prolonged neddylation of Cullin RING ligases (CRL), consequent to the inhibition of CSN5, resulted in degradation of IκBα and subsequent activation of the NF-κB pathway. This in turn promoted RhoB and, to a lesser extent, RhoA mRNA and protein synthesis, which resulted in increased activity of RhoGTPases, ROCK-mediated actin stress fiber formation, MLC phosphorylation and cell contraction.

Recently, we showed that general inhibition of CRL by MLN4924 enhanced RhoB expression and severly disrupted endothelial barrier function through the induction of RhoB-dependent cell contraction^9^. We identified the Cullin-3-Rbx1-KCTD10 complex as the ligase that mediates the poly-ubiquitination and degradation of RhoB^9^. Based on these findings, we hypothesized that cullin activation by CSN5i-3 would increase ubiquitination and degradation of RhoB, leading to improved endothelial barrier function. In contrast, however, prolonged Cullin-3 neddylation induced by CSN5i-3 resulted in decreased expression of Cullin-3, increased expression of RhoB and reduced endothelial integrity.

Several studies have shown that in endothelial cells RhoB protein levels are upregulated upon TNF-α stimulation of the NF-κB pathway, due to increased mRNA synhtesis^9,28,29^. This TNF-α-induced RhoB localizes to an endosomal compartment, in marked contrast to the pool of RhoB which accumulates following CRL inhibition and localizes to the plasma membrane^9^. Therefore, we analyzed localization of RhoB in HUVEC monolayers treated with CSN5i-3. To our surprise, we found that prolonged CRL neddylation leads to induction of RhoB expression within the endosomal compartment similar to the RhoB localization induced by TNF-α stimulation. Therefore we hypothesized that the NF-κB pathway was upregulated in endothelial cells treated with CSN5i-3. This assumption was further corroborated by the comparable induction of RhoB mRNA expression upon treatment with either CSN5i-3 or TNF-α.

Further analysis confirmed that the NF-κB pathway was indeed activated by CSN5i-3. Originally, the CSN5i-3 compound was designed to inhibit the COP9 metalloprotease in order to prolong Cullin activation^24^. The transition of Cullins between neddylated and non-neddylated states is required in order to exchange the substrate recognition receptors in the CRL complex. Schlierf *et al*. described that prolonged neddylation of Cullin RING ligases by CSN5 inhibition can lead to autodegradation of some but not all substrate recognition receptors. Interestingly, we found that the expression of Cullin proteins in endothelial cells can be affected by CSN5 inhibition as well. The associated deubiquitination activity of the CSN, which is now lost by inhibition of the complex using CSN5i-3, may contribute to the decreased stability and activity of some CRL complexes^19^. CSN5i-3 treatment reduced levels of Cullin-3, but did not significantly affect expression of Cullin-1. This suggests that prolonged CRL activation can have opposing effects on expression levels of substrates of different CRLs.

Comparable to our findings, Schweitzer *et al*. showed that knockdown of CSN2, also a component of the COP9 complex, leads to decreased expression of IκBα in HeLa cells, which eventually resulted in increased phosphorylated p65 in the nucleus after TNF-α stimulation^32^. The essential role of ubiquitination of IκB in the regulation of the NF-κB pathway was established previously^30^. Furthermore, the knockdown of CSN5 in endothelial cells increased NF-ĸB activity, ICAM-1 expression, and PMN adherence to the EC monolayer^18^. In the present study, in accordance with the previous work, we found that CSN5i-3 treatment decreased expression of IκBα and increased the phosphorylation of p65, resulting in increased ICAM-1 expression which promotes adhesion of PMNs.

The CSN5i-3 compound was found to be a promising candidate for potential treatment against cancer *in vitro* and in animal studies^24^. However, in our current study we found that prolonged neddylation of CRLs, induced by CSN5i-3, is not beneficial in endothelial cells, as it reduced barrier integrity and induced an inflammatory phenotype. The same effect on endothelial integrity was found upon general inhibition of CRLs by MLN4924, a compound which is already being tested in clinical trials^21–23^. In conclusion, our findings demonstrate that both prolonged activation, as well as -inhibition of CRLs can induce unwanted side effects, in the case of CSN5i-3 leading to endothelial inflammation and loss of endothelial barrier function. More specific inhibitors, targeting a smaller subset of E3 enzymes will be required to specifically modulate substrate degradation in cancer- and other cells and at the same time preserve the endothelial integrity and prevent adverse effects on the cardiovascular system.

## Methods

### Antibodies and reagents

The following antibodies were used in this study: αVE-cadherin XP (#2500), αRhoA (#2117), αRhoC (#3430), αCullin-3 (#2759), αp44/42 MAPK (ERK1/2) (#9102), αpMLC2 Ser19 (#3671), αGAPDH (#2118), αIκBα (#4841) and αpp65 (#93H1) (all from Cell Signaling Technology); αRhoB (#sc-8048 and #sc-180), αCullin-1 (#sc-17775), αICAM (#sc-8439) (all from Santa Cruz Biotechnology); αCullin-2 (#610778) and αp38 (#61268) (BD Transduction Laboratories) and αpp38 (#09–272) (Millipore).

Horseradish peroxidase-conjugated goat anti-rabbit and anti-mouse antibodies (Dako) were used as secondary antibodies for western blotting. For immunofluorescent staining DAPI (Thermo Fisher scientific), Alexa 488-secondary antibody (anti-rabbit and anti-mouse, Invitrogen) and Acti-stain 670 phalloidin (Cytoskeleton) were used.

The following inhibitors and cytokines were used in this study: CSN5i-3 (Novartis), MLN4924 (Active Biochem), Y27632 (#Y0503) (Sigma), TNF-α (#300-01 A) (PeproTech), BAY11-7085 (Cayman Chemical).

### Cell culture

#### Freshly isolated HUVECs

Primary Human Umbilical Vein Endothelial Cells (HUVECs) were isolated from umbilical cords of healthy donors. Umbilical cords were provided by the Amstelland Ziekenhuis, Amstelveen. Informed consents were obtained from all donors in accordance with the institutional guidelines and the Declaration of Helsinki. The cells were isolated and characterized as described by Jaffe *et al*.^33^. The primary HUVECs were cultured in M199 medium supplemented with: penicillin 100 U/mL and streptomycin 100 μg/mL, L-glutamine 2 mMol/L (all from Bio Whittaker/Lonza), heat-inactivated human serum 10% (Sanquin, Amsterdam, The Netherlands), heat-inactivated new-born calf serum 10% (Gibco), crude endothelial cell growth factor 150 μg/mL (locally prepared from bovine brains) and heparin 5 U/mL (Leo pharmaceutical products, Weesp, The Netherlands). Cells were cultured at 37 °C and 5% CO_2_, and medium was refreshed every second day. For all experiments, pools of HUVECs of 3 donors in passages 1–2 were used.

#### Lonza HUVECS

Primary HUVECs were purchased from Lonza (#CC-2519) and cultured on fibronectin-coated plates in Endothelial Cell medium (ECM), supplemented with singlequots (Sciencell Research Laboratories). Cells were cultured at 37 °C and 5% CO_2_ and the medium was refreshed every second day. Experiments were performed with cells until passage 7.

#### HEK293T cells

HEK293T cells (ATCC) (#CRL-3216) were grown in Dulbecco’s Modified Eagle Medium (Gibco) (#41966-029) supplemented with penicillin 100 U/mL and streptomycin 100 μg/mL, L-glutamine 2 mMol/L (all from Bio Whittaker/Lonza), 1 mM sodium pyruvate (Gibco) (#11360-070) and 10% Fetal Bovine Serum (PAA) (#A15-101).

### siRNA transfection

One day prior to transfection HUVECs were seeded on fibronectin coated 12-well cell culture plates. On the day of transfection cells were 70–80% confluent. Transfection was performed using Dharmafect I reagent (Dharmacon/Horizon discovery) and 20 nM of one or combination several of the following siRNAs: ON-TARGET plus Human RHOA siRNA-SMART pool, ON-TARGET plus Human RHOB siRNA-SMART pool or ON-TARGET plus Human RHOC siRNA-SMART pool. ON-TARGET plus Non-targeting Control pool was used as a negative control. Transfected cells were used for experiments at 72 hours post-transfection.

### Protein analysis

The cells were stimulated with compounds for indicated times as stated in individual experiments. To analyze protein expression, cells were washed with serum- and growth factor- deprived medium and whole-cell lysates were collected in 2x SDS sample buffer (SB) (125 mM Tris-HCl, 4% SDS, 20% glycerol, 100 mM DTT, 0.02% Brom Phenol Blue in MilliQ). Protein samples were separated using SDS-PAGE and transferred to nitrocellulose membranes, followed by incubation with designated primary antibodies. Protein bands were visualized with enhanced chemiluminescence (Amersham/GE-healthcare) on the AI600 machine (Amersham/GE-healthcare).

### RhoGTPase activation assay

Confluent endothelial cells seeded on 60 cm^2^ dishes were treated with 1 or 4 uM CSN5i-3 for 5 hours. After treatment the cells were washed with ice cold PBS and lysed on ice. The lysates were tested for Rho A, -B and -C activity using Rho Activation Assay Biochem Kit^TM^ (Cytoskeleton) according to the manufacturer’s instructions.

### Endothelial barrier function assays

Endothelial barrier function was measured with electrical cell-substrate impedance sensing (ECIS) and passage of Horse Radish Peroxidase (HRP). For ECIS measurements, cells were seeded on gelatin-coated ECIS plates containing gold intercalated electrodes (Applied biophysics). When primary isolated HUVECs were confluent, they were serum-starved in M199 medium supplemented with 1% human serum albumin (HSA, Sanquin) for approximately 90 minutes. Subsequently, compounds were added to the cell medium. For Lonza HUVECs, ECIS plates were coated with 5 μg/ml Fibronectin and the compounds were directly added to the ECM medium.

Macromolecular permeability of the endothelial barrier was measured by passage of horse radish peroxide (HRP) through human endothelial barriers. Lonza HUVECs were seeded on top of gelatin-fibronectin coated Thin-Certs^TM^ (Greiner Bio-One) and cultured in ECM with a medium change every second day. When a stable barrier was formed the medium in the upper compartment was replaced by complete medium containing HRP 5 μg/mL and CSN5i-3 or a vehicle control. At several time-points a sample was taken from the lower compartment. The HRP concentration was calculated by measuring absorbance after adding tetramethylbenzidine (TMB) (Upstate/Millipore) and sulfuric acid to stop the reaction.

### Immunofluorescence imaging of cultured endothelial cells

Lonza HUVECs were seeded on 2 cm^2^ coverslips (Thermo Scientific, Menzel-gläser) (#10319303) which were pre-coated with 5 μg/ml fibronectin. Cells were grown until confluency with a medium change every second day and upon reaching confluency, experiments were performed. Cells were fixed with warm (37 °C) 4% paraformaldehyde (Sigma Aldrich) (#158127) in phosphate buffered saline (PBS) (B Braun) (#3623140) and incubated at room temperature for 15 minutes. The PFA was washed away with PBS, cells were permeabilized with 0,2% triton X-100 in PBS for 3 minutes and blocked for 30 minutes with 1% HSA in PBS. Hereafter, coverslips were stained with primary antibodies in 1% HSA/PBS for 1 hour at room temperature or overnight at 4 °C. After washing with PBS, coverslips were incubated with a FITC-labeled secondary antibody (anti-rabbit or anti-mouse 1:100 in 1% HSA/PBS), Acti-stain 670 phalloidin (Cytoskeleton) (#PHDN1-A) and DAPI (Thermo Fisher Scientific) for 1 hour at room temperature. Coverslips were mounted with Mowiol4-88/DABCO solution (Calbiochem, Sigma Aldrich). Confocal scanning laser microscopy was performed on a Nikon A1R confocal microscope (Nikon). Images were analyzed and equally adjusted with ImageJ software.

### RT-PCR

HUVECs (Lonza) were treated with CSN5i-3 or TNF-α for 5 hours. Total RNA was purified from the HUVECs using TRIzol (Thermo Fisher Scientific) and Direct-zol RNA MiniPrep kit (Zymo Research) and 500 ng of RNA was used for cDNA synthesis using iScript cDNA synthesis kit (Bio-Rad). Quantitative real time PCR was performed (iQ SYBR Green Supermix, Bio-Rad) with the CFX384 Real-Time system (Bio-Rad). Following primers were used for amplification: TGCACCACCAACTGCTTAGC-3′ (GAPDH-F) and 5′-GGCATGGACT GTGGTCATGAG-3′ (GAPDH-R) for GAPDH, 5′-GAGGTGGATGGAA AGCAGGTAGAGTTG-3′ (RhoA-F) and 5′-TTTCACCGGCTCCTGCTTCATCTTGG-3′ for (RhoA-R) for RhoA, 5′-AGA CGTGCCTGCTGATCGTGTTCAG-3′ (RhoB-F) and 5′-CACATTGGGACAGAAGTGCTTCACC-3′ (RhoB-R) for RhoB.

### PMN isolation

Polymorphonuclear neutrophils were isolated from fresh heparinized blood obtained from healthy donors. The blood was diluted 1:1 with PBS layered onto Lymphoprep (#07801) (Stem cell technologies). By centrifugation, blood components were separated and all the layers except the layer containing erythrocytes, neutrophils and eosinophils were removed. After erythrocyte lysis in cold lysis buffer (155 mM NH_4_Cl, 10 mM KHCO_3_, 0.1 mM Na-EDTA, pH 7.4), PMNs were washed twice with PBS and resuspended in HEPES buffer (20 mM HEPES, 132 mM NaCl, 6 mM KCl, 1 mM MgSO_4_, 1.2 mM K_2_HPO_4_, 1 mM CaCl_2_, 0.5% Human Serum Albumin (HSA, Sanquin). PMNs were stored at room temperature for a maximum of 3 hours upon isolation.

### PMN transendothelial migration

Lonza HUVECs were grown on 2 cm^2^ fibronectin-coated wells until confluency. Freshly isolated PMNs were labelled for 10 minutes at 37 °C with 1 μg/ml calcein AM (#C3099) (Thermo Fischer Scientific), washed once with HEPES buffer and incubated for another 30 minutes at 37 °C. A total of 500,000 PMNs were added per 2 cm^2^ well on a CSN5i-3 or TNF-α treated HUVEC monolayer and placed in the incubator at 37 °C and 5% CO_2_. After 25 minutes, non-adherent PMNs were washed away with HEPES buffer and cells were fixed. Adherent PMNs were visualized using the green fluorescence filter of a LS720 Microscope (Etaluma). Treatments were performed in duplicate and the number of adherent PMNs per field of view was counted from five random fields per well using ImageJ.

### Zebrafish husbandry, embryo care and compound treatment

Adult *Tg*(*Fli1:GFP*)^*y1*^*casper* zebrafish were maintained at 26 °C in aerated 5-L tanks with a 10/14 hour dark/light cycle^34,35^. The *Tg(fli1:GFP)*^*y1*^ zebrafish line expresses GFP in the endothelial cells of the entire vasculature under the control of the fli1 promoter. Zebrafish were raised, staged and maintained according to standard procedures (zfin.org). Zebrafish embryos were collected within the first hours post fertilization (hpf) and kept at 28 °C in E3-medium (5.0 mM NaCl, 0.17 mM KCl, 0.33 mM CaCl·2H_2_O, 0.33 mM MgCl_2_·6H_2_O) supplemented with 0.3 mg/L methylene blue. For compound treatment, zebrafish embryos were manually dechorionated at 24 hpf and transferred to separate wells. After experiments were performed zebrafish embryos were anesthetized in 0.02% (*w*/*v*) buffered 3-aminobenzoic acid methyl ester (pH 7.0) (Tricaine) (Sigma-Aldrich) (#A5040) and euthanized by hypothermic shock. All experiments involving zebrafish embryos were according to local animal welfare regulations. VU University medical center animal welfare committee approved the breeding procedure of the adult zebrafish. Experimental procedures were performed in zebrafish larvae from 1–4 days post-fertilization prior to the stage of free living, which is in accordance with the EU Animal Protection Directive 86/609 EEC.

### Dextran leakage assay in Zebrafish embryos

At 24 hpf, CSN5i-3 was added to the water of *Tg(fli1:GFP)*^*y1*^ *casper* zebrafish embryos to treat them for 48–72 hours with the compound. Zebrafish embryos were subsequently injected with ~1 nl of a 2 mg/ml solution of 70 kDa TMR dextran (#D1818) (Thermo Fisher Scientific) into the vasculature at the intersection of the common cardinal vein, the posterior cardinal vein and the primary head sinus using a Pneumatic PicoPump (#SYS-PV820) (World precision instruments). During injection and imaging, the zebrafish embryos were anaesthetized in 0.02% (*w*/*v*) buffered 3-aminobenzoic acid methyl ester (pH 7.0) (Tricaine) (#A5040) (Sigma-Aldrich). For live imaging, zebrafish embryos were mounted in an uncoated 8-well μ-slide (#80827) (Ibidi) in 1.5% low melting point agarose dissolved in egg water (60 μg/mL sea salts (Sigma-Aldrich; S9883) in MilliQ) with addition of 0.02% (*w*/*v*) buffered 3-aminobenzoic acid methyl ester (pH 7.0) (Tricaine) (#A5040) (Sigma-Aldrich). Zebrafish embryos were imaged after 20 minutes using a Zeiss wide field microscope at 10x magnification.

### Preparation of zebrafish embryo lysates for western blot

For western blot analysis of Cullin-3 expression, whole lysate of the zebrafish embryos was prepared between 72 hpf and 96 hpf, the same time-frame in which the dextran was injected for analysis of leakage. Prior to lysis, zebrafish embryos were anesthetized in 0.02% (*w*/*v*) buffered 3-aminobenzoic acid methyl ester (pH 7.0) (Tricaine) (Sigma-Aldrich) (#A5040), collected in an Eppendorf tube and euthanized by hypothermic shock. The water was removed and 10 ul 2x SDS sample buffer per fish was added. The lysate was boiled for 3 minutes at 95 °C and homogenized by sonication.

### Caspase-3/7 assay

HUVECs were grown to confluency in a Black Falcon 96-well plate with clear bottom. For the analysis of caspase-3/7 activity, medium was replaced by fresh medium containing 0, 1 or 4 μM CSN5i-3. Cells were treated for 5 hours with 200 nM Staurosporin as a positive control. After 5 and 24 hours of treatment with CNS5i-3, caspase-3/7 activity was analyzed using the Apo-ONE® Homogeneous Caspase-3/7 Assay kit and following the manufacturer’s protocol.

### RhoB ubiquitination assay

HEK293T cells were co-transfected with mCherry-RhoB and HA-Ubiquitin using TransIT-LT1 (Mirus) (#MIR 2300) and following the manufacturer’s protocol. The next day, cells were treated for five hours with 1 or 4 μM CSN5i-3 or 500 nM MLN4924, with addition of 2.5 μM MG132 for the last four hours. Next, denaturing HA-immunoprecipitation was performed as described previously^36^.

### Statistical analysis

Data is represented as mean ± SD. Statistical significance was tested by one-way ANOVA or repeated measures ANOVA with Dunnet’s post-hoc test, unless indicated differently. P-values were considered statistically significant if p < 0.05. Analysis was performed using GraphPad Prism 7 software.

## Supplementary information

Supplemental information
