# Supplementary material for: CSN5 inhibition triggers inflammatory signaling and Rho/ROCK-dependent loss of endothelial integrity
Source: Sci Rep. 2019 May 31;9:8131. doi: 10.1038/s41598-019-44595-4 (PMC6544660; doi:10.1038/s41598-019-44595-4)

CSN5 inhibition triggers inflammatory signaling and Rho/ROCK-dependent loss of endothelial integrity

Jisca Majolée[1¶], Manon C.A. Pronk[1¶], Kin K. Jim[2], Jan S.M. van Bezu[1], Astrid M. van der Sar[2], Peter L. Hordijk[1], Igor Kovačević[1*]

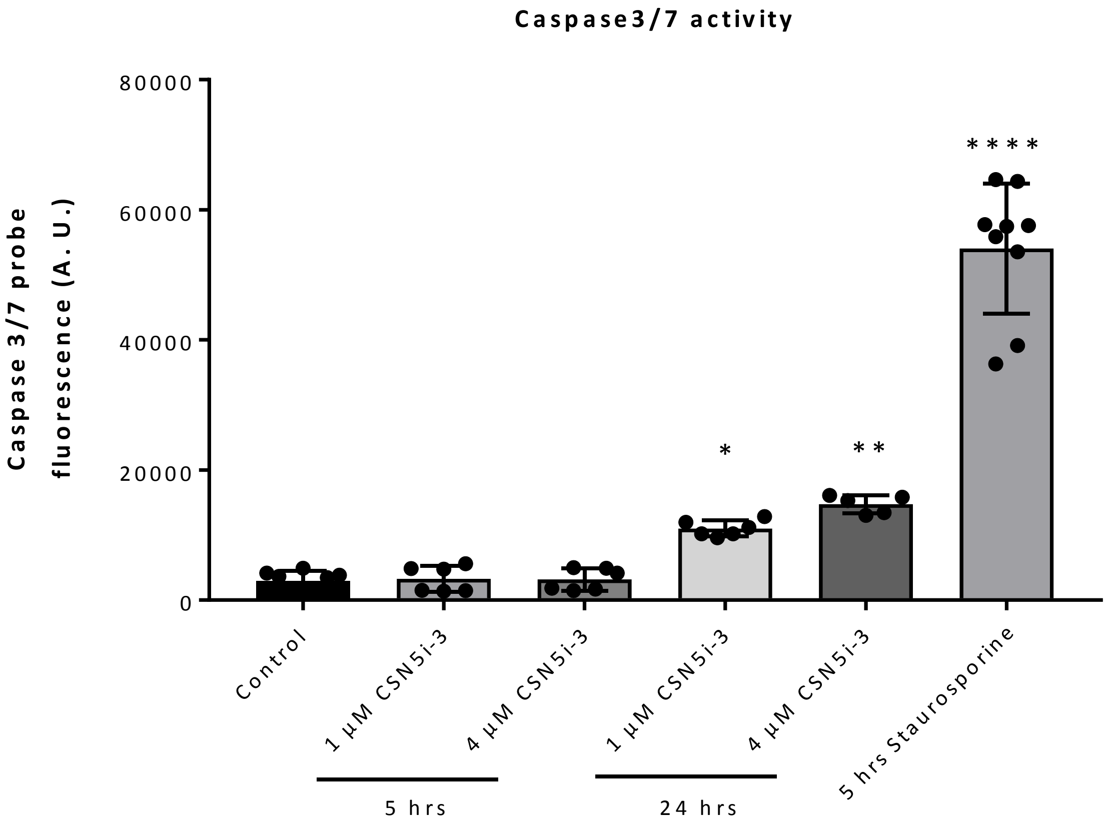

Supplemental Figure 1 – CSN5i-3 induces apoptosis after 24 hours, but not after 5 hours of treatment.

Caspase-3/7 assay of HUVEC monolayers treated with CSN5i-3 (1 or 4 µM) for 5 or 24 hours or 200 nM Staurosporin for 5 hours as a positive control. * $p<0.05$, ** $p<0.01$, **** $p<0.0001$ compared to control after Dunnet post-hoc analysis of one-way ANOVA.

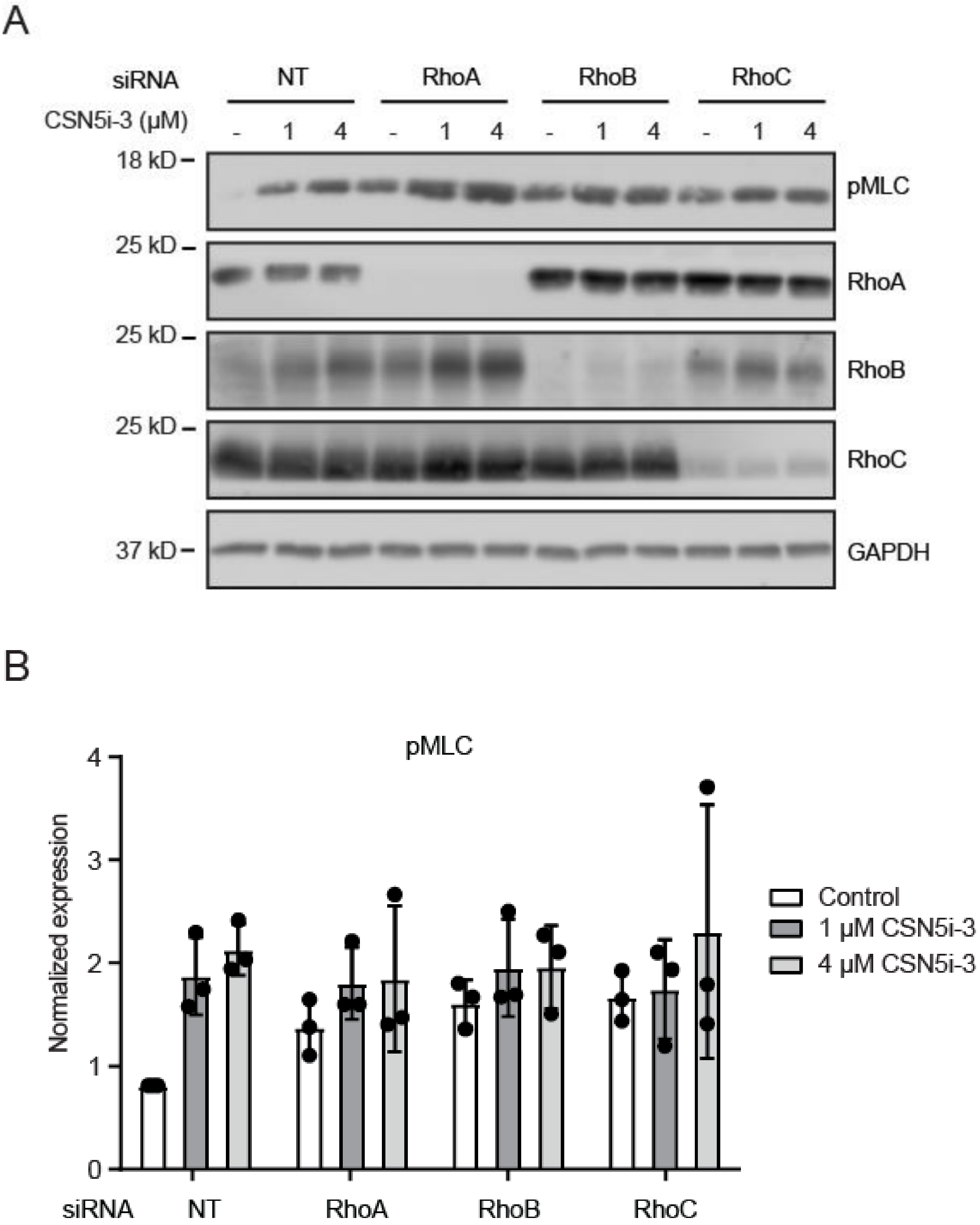

Supplemental Figure 2 – single knockdown of RhoA, RhoB or RhoC does not effectively prevent MLC phosphorylation by CSN5i-3.

A) Western blot analysis of pMLC, RhoA, RhoB, and RhoC expression in RhoA, RhoB and RhoC knockdown cells after 5 hr of CSN5i-3 (1 μM or 4 μM) treatment. GAPDH was used as loading control. Blot images were cropped for clarity of presentation. B) Quantification of pMLC levels compared to control.

Supplemental Figure 3 – full blots of Figure 1D

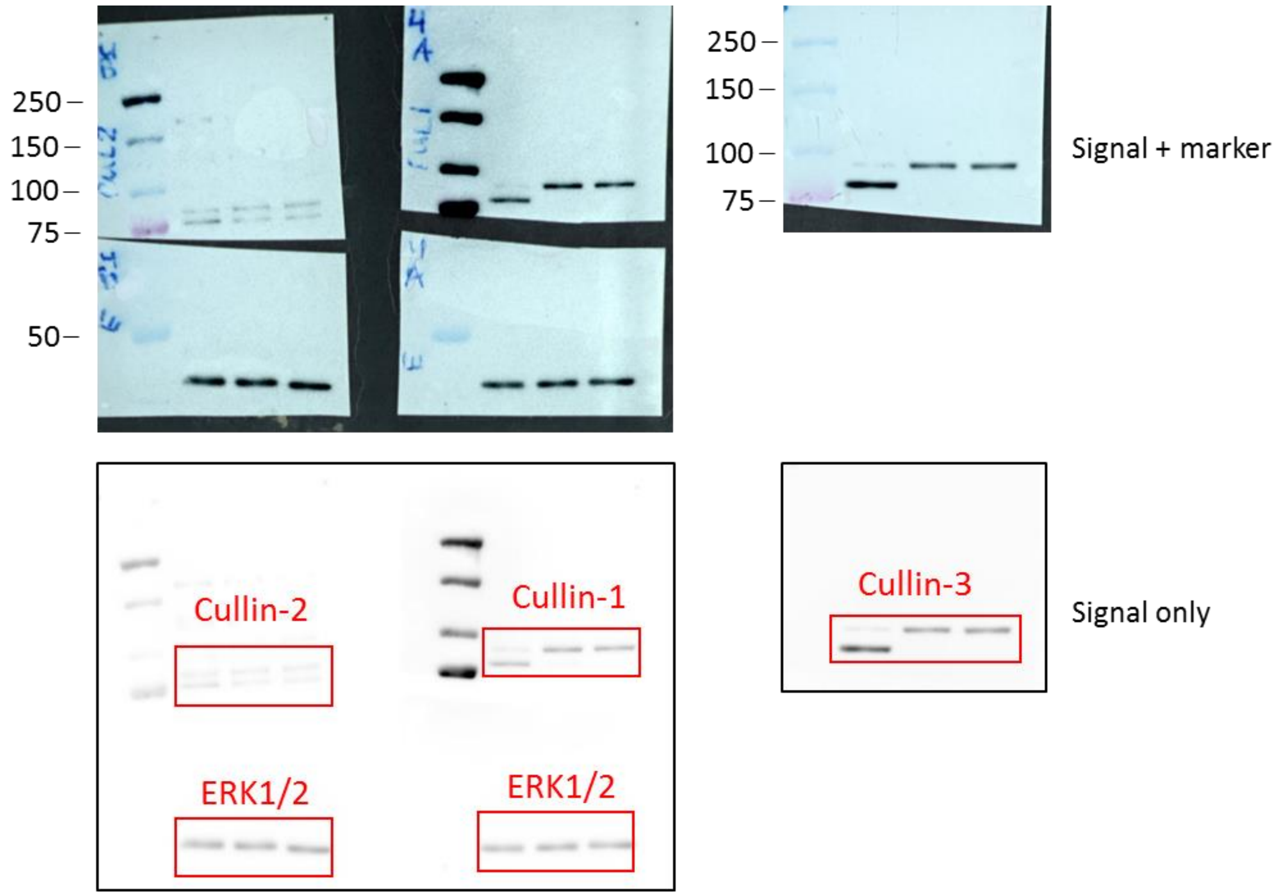

Supplemental Figure 4 – full blots of Figure 2ABC

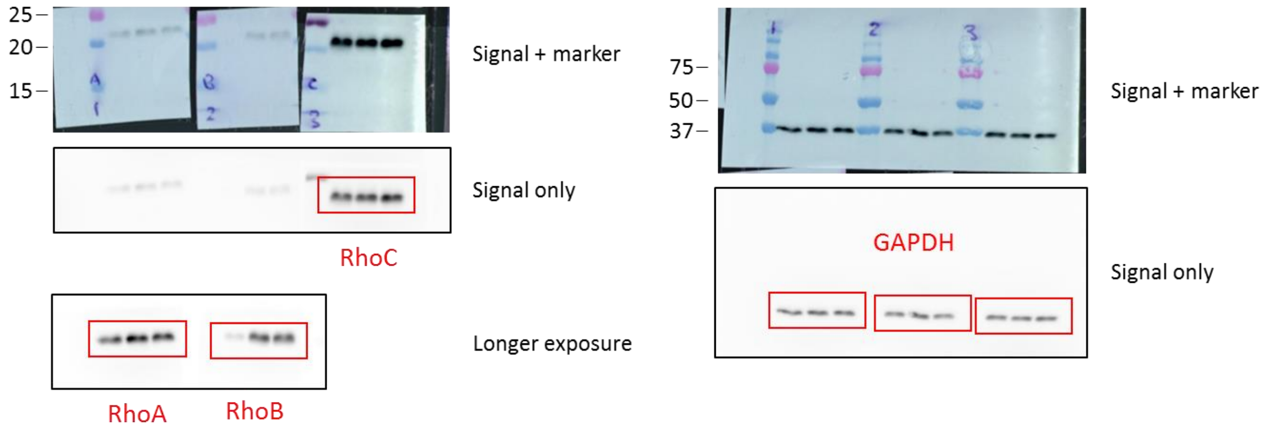

Supplemental Figure 5 – full blots of Figure 2E

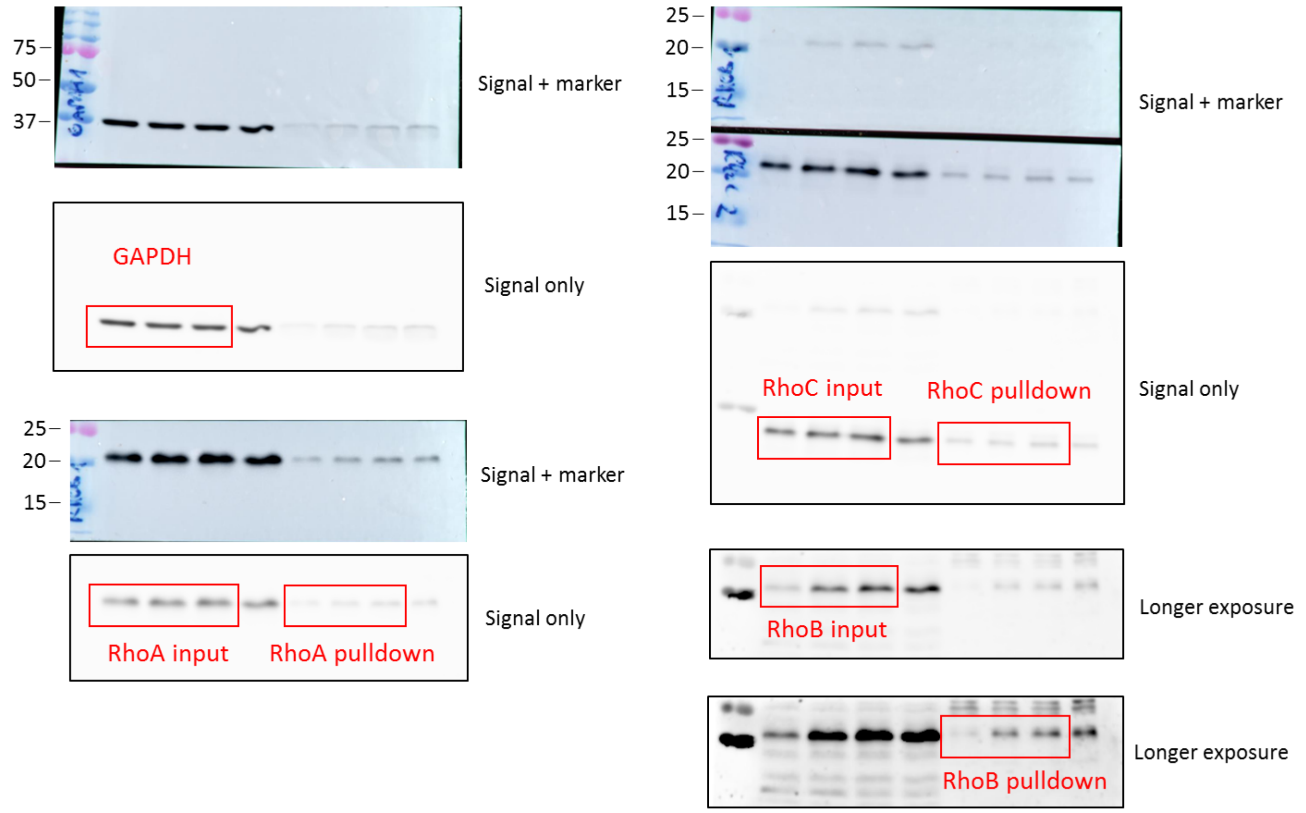

Supplemental Figure 6 – full blots of Figure 3D

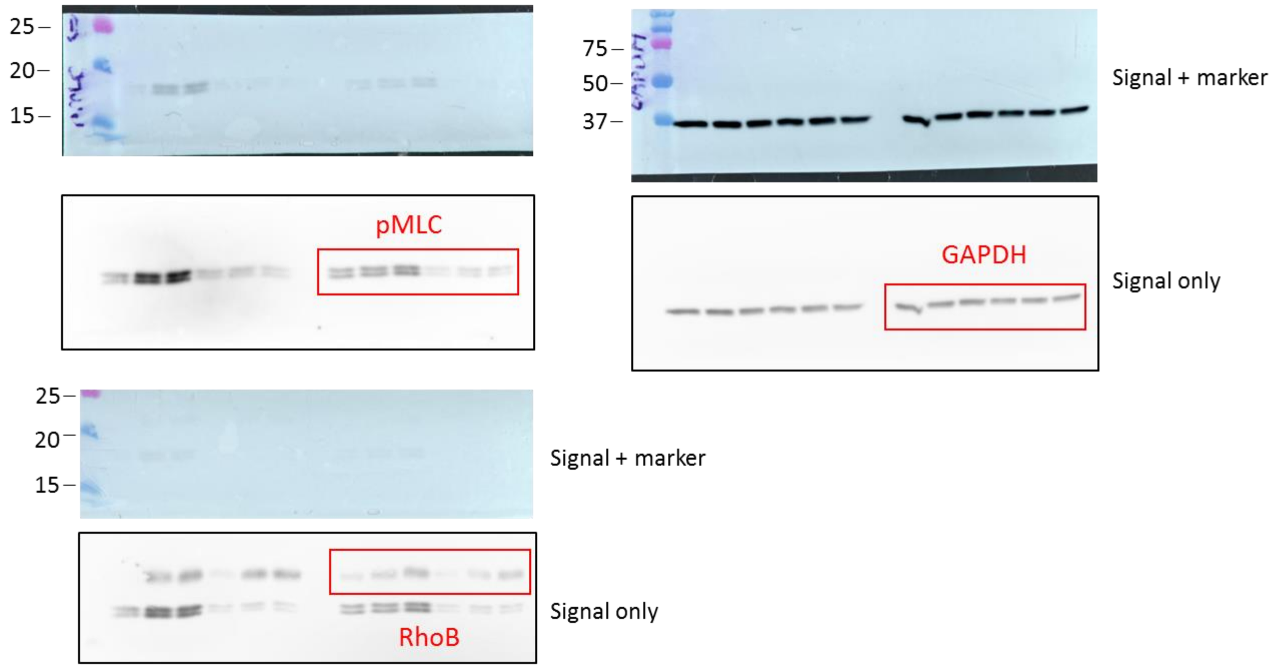

Supplemental Figure 7 – full blots of Figure 3G

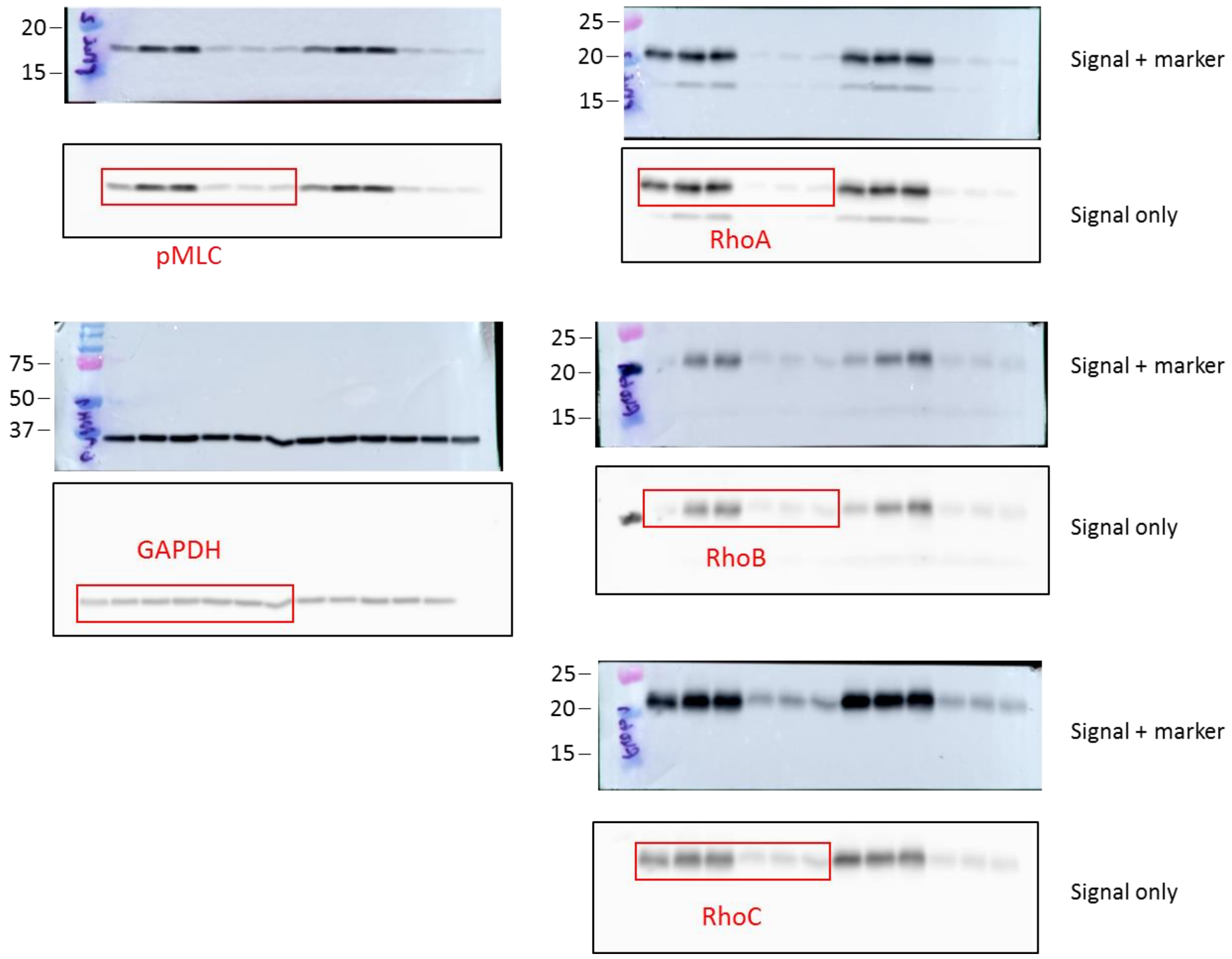

Supplemental Figure 8 – full blots of Figure 4A

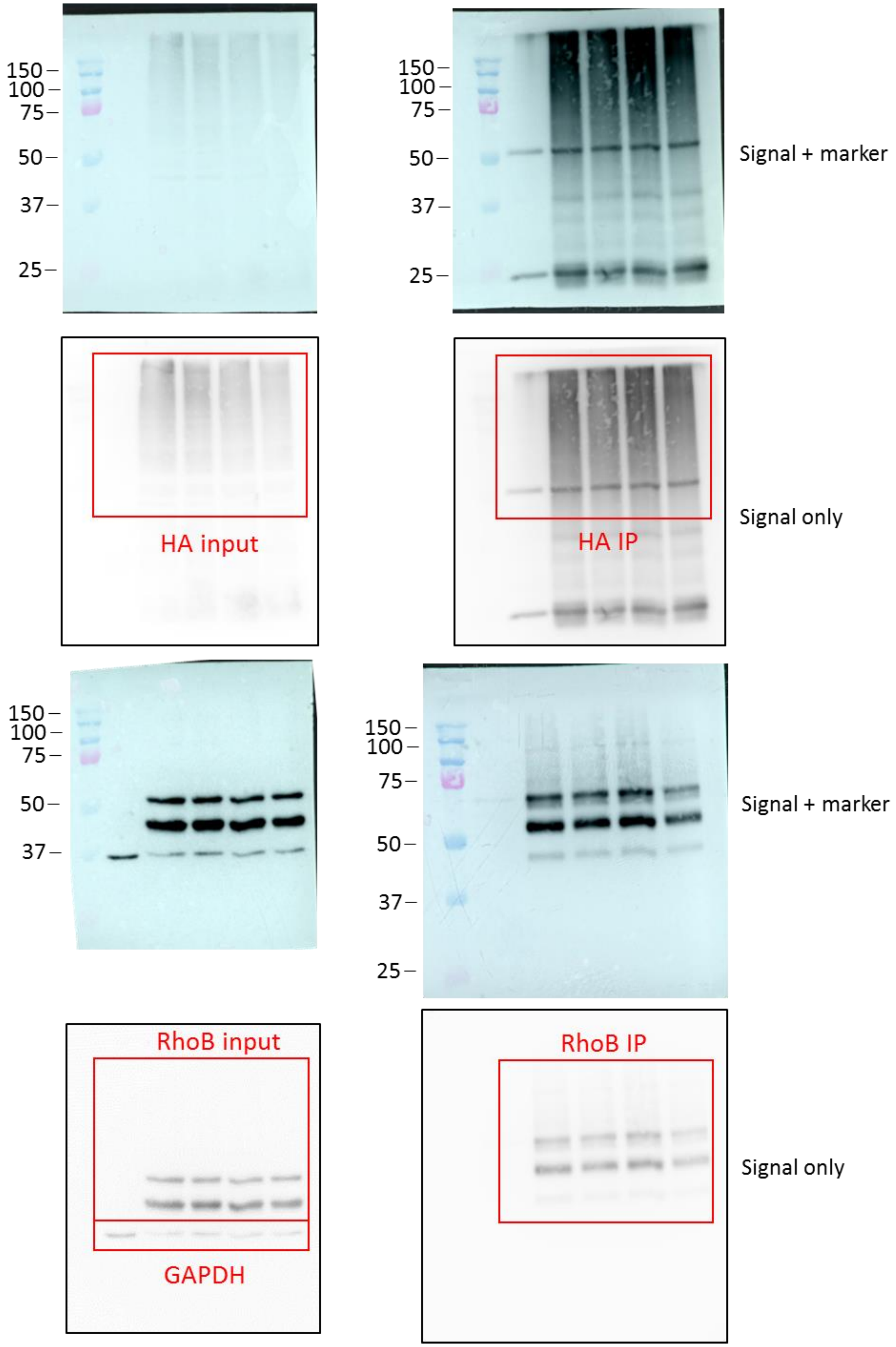

Supplemental Figure 9 – full blots of Figure 4D

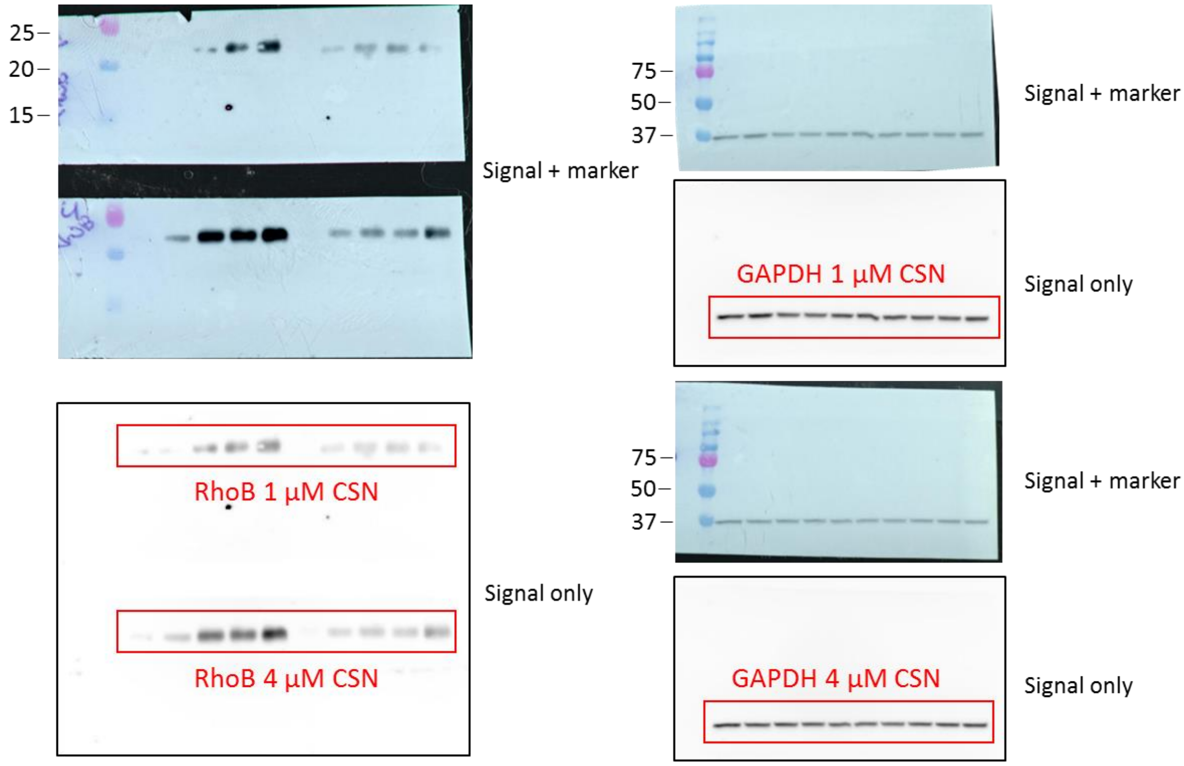

Supplemental Figure 10 – full blots of Figure 5A

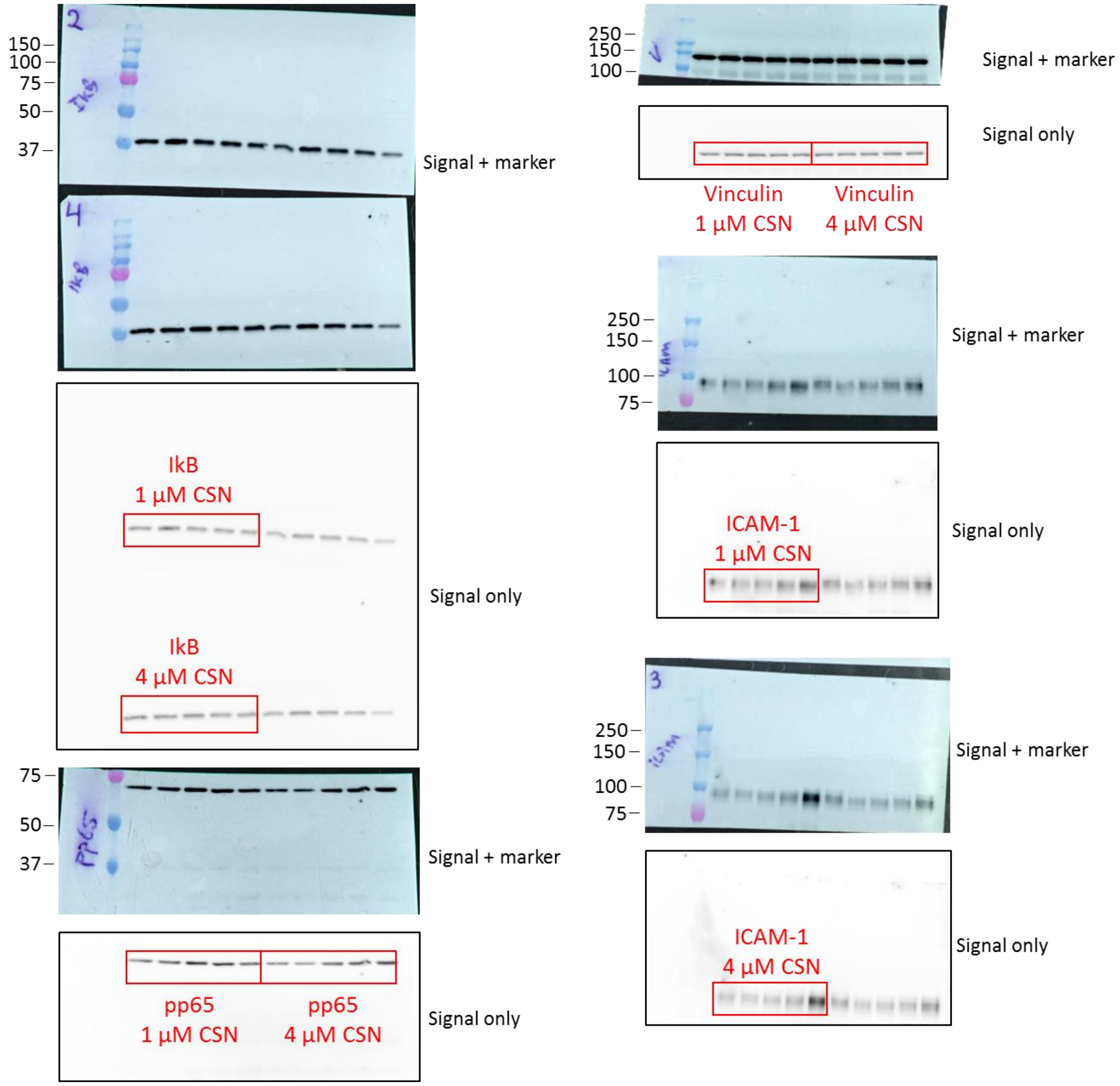

Supplemental Figure 11 – full blots of Figure 5E

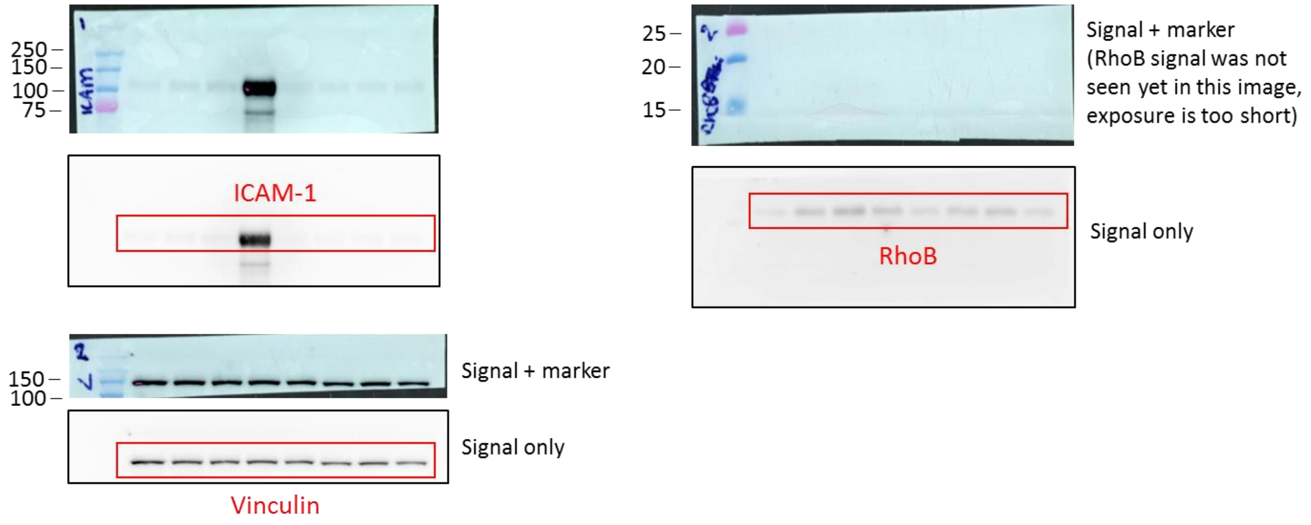

Supplemental Figure 12 – full blots of Figure 6A

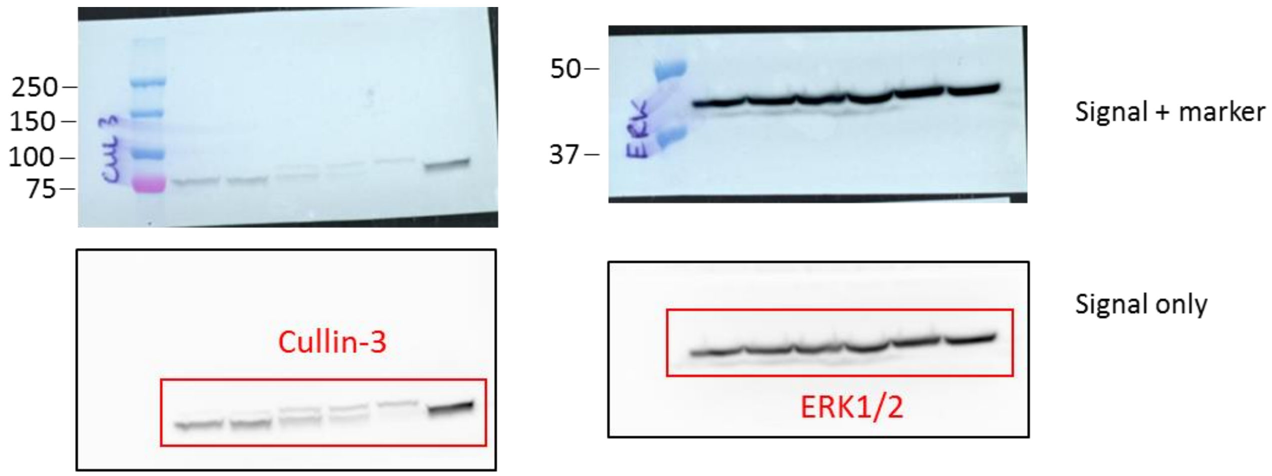

Supplement: Supplementary file 1 — Supplemental information [file 41598_2019_44595_MOESM1_ESM.pdf]
